# Pharmacokinetics and analgesic effects of intravenous, intramuscular or subcutaneous buprenorphine in dogs undergoing ovariohysterectomy: a randomized, prospective, masked, clinical trial

**DOI:** 10.1186/s12917-020-02364-w

**Published:** 2020-05-24

**Authors:** Paulo Vinicius Steagall, Hélène Louise Marcelle Ruel, Tomoyo Yasuda, Beatriz Paglerani Monteiro, Ryota Watanabe, Marina Cayetano Evangelista, Francis Beaudry

**Affiliations:** 1 Department of Clinical Sciences, Faculty of Veterinary Medicine, Université de Montréal, Saint-Hyacinthe, QC Canada; 2 Diplomate American College of Veterinary Anesthesia and Analgesia, 3200 rue Sicotte, Saint-Hyacinthe, QC J2S 2M2 Canada; 3 Department of Veterinary Biomedical Sciences, Faculty of Veterinary Medicine, Université de Montréal, Saint-Hyacinthe, QC Canada

**Keywords:** Analgesia, Buprenorphine, Canine, Pain, Ovariohysterectomy, Simbadol, Pharmacokinetics

## Abstract

**Background:**

Buprenorphine is used for canine postoperative pain management. This study aimed to describe the pharmacokinetics and evaluate the analgesic efficacy of buprenorphine (Simbadol, 1.8 mg/mL) administered by different routes in dogs undergoing ovariohysterectomy. Twenty-four dogs were included in a randomized, prospective, masked, clinical trial. Buprenorphine (0.02 mg/kg) was administered intravenously (IV), intramuscularly (IM) or subcutaneously (SC) (*n* = 8/group) 0.5 h before general anesthesia with propofol-isoflurane. Carprofen (4.4 mg/kg SC) was administered after anesthetic induction and before ovariohysterectomy. Pain was scored using the short-form Glasgow composite pain scale for dogs (SF-GCPS). Dogs were administered morphine (0.25 mg/kg IV) when SF-GCPS scores were ≥ 5/20. Blood sampling was performed up to 720 min after drug administration. Plasma buprenorphine and norbuprenorphine concentrations were analyzed using liquid chromatography mass spectrometry. Pharmacokinetics of buprenorphine was described using a non-compartmental model (PK Solver 2.0). Statistical analysis was performed using linear mixed models and Fisher’s exact test (*p* < 0.05).

**Results:**

Pain scores were significantly higher than baseline after IV (0.5–2 h), IM (0.5–3 h) and SC (0.5–4 h) but not among groups. Prevalence of rescue analgesia was significantly higher in SC (7/8 dogs) than IV (2/8) but not different between IV and IM (3/8) or IM and SC. The frequency of rescue analgesia was not significantly different among groups (IV = 2, IM = 5 and SC = 9). Norbuprenorphine was not detected. For IV, IM and SC administration, clearance was 1.29, 1.65 and 1.40 L/hour/kg, volume of distribution was 6.8, 14.2 and 40.1 L/kg, the elimination half-life was 3.7, 5.7, 22 h, and the area under the plasma concentration-time curved extrapolated to infinity was 15.7, 12.4 and 16.4 ng/mL/hour, respectively. Bioavailability for IM and SC was 62.6 and 40%, respectively. Maximum plasma concentrations of buprenorphine were 6.2 and 1.3 ng/mL at 0.14 and 0.33 h after IM and SC administration, respectively.

**Conclusions:**

The route of administration influences the analgesic efficacy of buprenorphine in dogs. SC administration of buprenorphine failed to provide clinical analgesia due to erratic drug absorption. At the doses administered, the IV and IM routes are preferred for postoperative analgesia.

## Background

Buprenorphine is a partial agonist of μ-opioid receptors used for perioperative pain control as part of multimodal analgesia in dogs and cats [1–4]. The pharmacokinetics (PK) of buprenorphine using different routes of administration have been described in dogs and cats [3, 5–9]. However, to the authors’ knowledge, the pharmacodynamic effect of buprenorphine has not always been reported in these studies especially in the presence of anesthetics and during clinical conditions.

The American Veterinary Medical Association (AVMA) and the veterinary profession in the United States of America are concerned with an ongoing opioid shortage due to the national response to human opioid addiction. In brief, there has been a shortage of full agonists of μ-opioid receptors that are used “off-label” in veterinary pain management. This issue has compromised patient care and veterinarians do not always have means to effectively manage pain.^1^ A high-concentration formulation of buprenorphine (Simbadol™, buprenorphine injection, 1.8 mg/mL) is licensed by the Food and Drug Administration to provide postoperative pain relief in cats for up to 3 days when administered at 0.24 mg/kg subcutaneously (SC) every 24 h. Considering the opioid shortage, there is now an interest in using Simbadol for postoperative pain as part of multimodal analgesia in dogs.

A recent study demonstrated that the intramuscular (IM) administration of buprenorphine (0.02 mg/kg) in combination with carprofen (4.4 mg/kg SC) provided effective postoperative analgesia in dogs undergoing ovariohysterectomy [4]. Pain scores were not significantly different after the administration of similar doses of two different concentrations of buprenorphine (Simbadol and Vetergesic 0.3 mg/mL). However, the prevalence of rescue analgesia between treatments (25% with Vetergesic versus 0% with Simbadol) was of clinical relevance since the observed analgesic effect was large, albeit not statistically significant [4]. Therefore, a pharmacokinetic study would be helpful to understand the absorption, distribution, metabolism and elimination of Simbadol in dogs. Ideally, this should be described in a comparative manner using three common routes of administration (i.e. IV, IM and SC) since this has been shown to influence the analgesic efficacy of buprenorphine in cats [3, 9, 10]. The study should also reflect the clinical PK of the drug in patients undergoing general anesthesia/surgery with simultaneous postoperative pain assessment (i.e. pharmacodynamic effect).

The hypotheses of this study were that the IV and IM concentration-time profile would decrease curvilinearly whereas the SC PK would show erratic drug absorption after the administration of buprenorphine, and that the route of administration would influence the postoperative pain scores and prevalence of rescue analgesia, similarly to the previous findings in cats [9]. The aims of this study were to describe the PK and to evaluate the analgesic efficacy of Simbadol after IV, IM and SC administration in dogs undergoing ovariohysterectomy receiving carprofen.

## Results

**Fig. 1:**
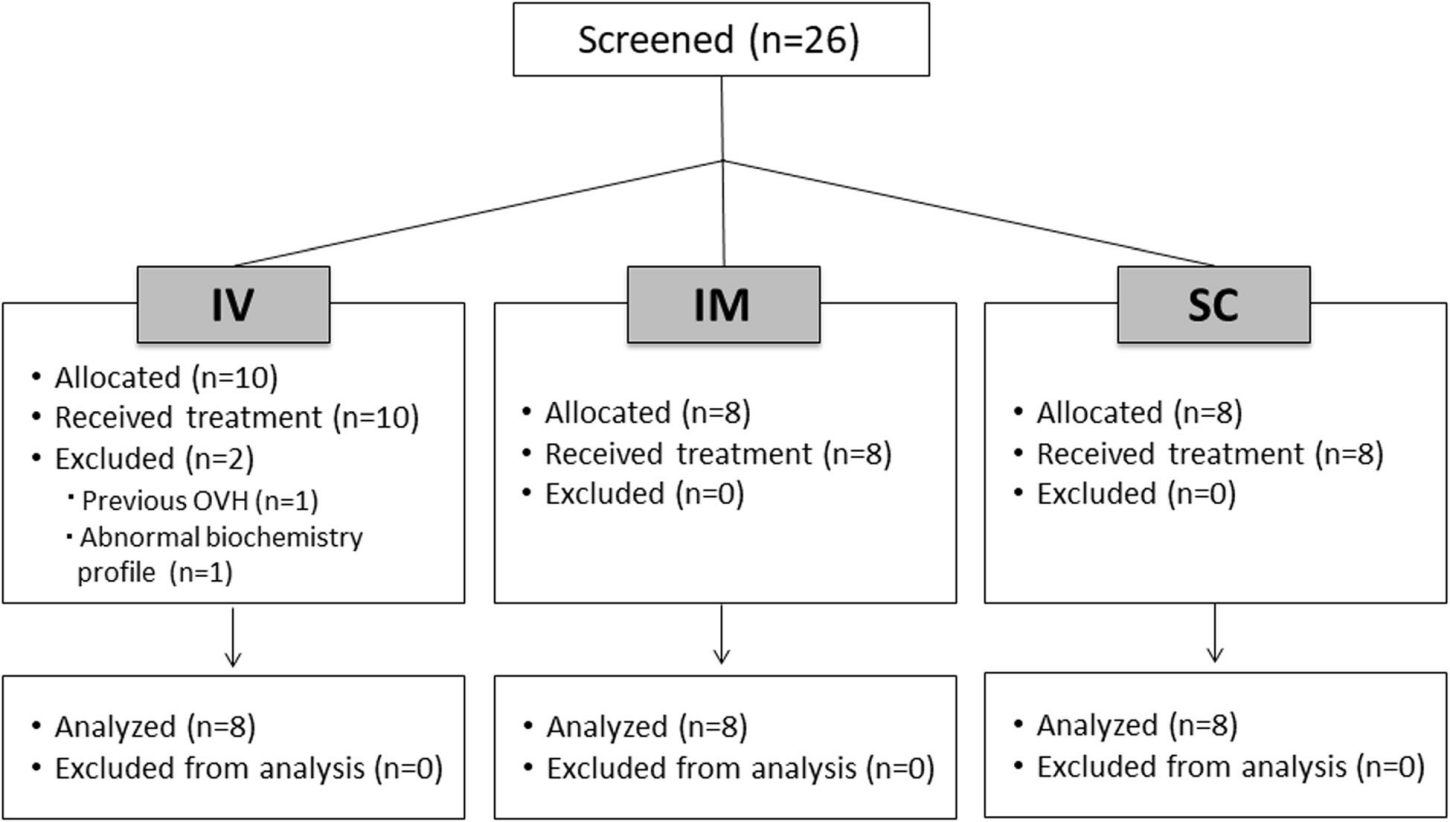
CONSORT flow diagram with the number of patients screened and allocated to each treatment group according to the study randomization

Twenty-six dogs were enrolled and 24 were included in the study (Fig. 1). Table 1 presents demographic data, doses of propofol, duration of anesthesia and surgery, time to extubation, and length of incision in each group and whether there were any significant differences between groups for these variables. Dog breeds in each group were as follows: IV [Mixed Breed (*n* = 2), German Shepherd (*n* = 2), Labrador Retriever (*n* = 1), American Bulldog (*n* = 1), Pug (*n* = 1) and American Staffordshire (*n* = 1)]; IM [Mixed breed (*n* = 2), Labrador Retriever (*n* = 2), Shetland Sheepdog (*n* = 1), Pit Bull (*n* = 1), Miniature Pinscher (*n* = 1), Siberian Husky (*n* = 1)] and SC [Labrador Retriever (*n* = 3), Mixed Breed (*n* = 2), Boston Terrier (*n* = 2), German Shepperd (*n* = 1)]. All dogs were discharged approximately 36 h after surgery.

**Table 1:** Demographic data, doses of propofol, duration of anesthesia and surgery and length of incision in dogs undergoing ovariohysterectomy and administered buprenorphine (Simbadol; 0.02 mg/kg) by the intravenous (IV), intramuscular (IM) or subcutaneous (SC) routes of administration

Variables	IV *n* = 8	IM *n* = 8	SC *n* = 8	*p value*
Body condition score (1–9)	5 (5–8)	5 (4–7)	5 (4–7)	0.4127
Body weight (kg)	22.4 ± 9.6	19.0 ± 4.3	18.2 ± 7.2	0.4921
Age (year)	4.6 ± 2.5^a^	2.9 ± 2.4	1.7 ± 1.8^b^	*0.0489*
Propofol (mg/kg)	4.2 ± 0.8^a^	4.6 ± 0.7	5.3 ± 1.0^b^	*0.0478*
Duration of anesthesia (minutes)	52.6 ± 9.7	47.4 ± 8.6	44.3 ± 6.8	0.1597
Duration of surgery (minutes)	33.9 ± 8.8	30.3 ± 6.7	26.6 ± 3.9	0.1280
Time to extubation (minutes)	3.1 ± 1.6	2.4 ± 1.1	2.3 ± 0.9	0.3087
Length of incision (cm)	3.9 ± 1.4	4.1 ± 1.3	3.6 ± 1.0	0.7093

Data are presented as mean ± standard deviation or as mean (range) where appropriate. The *p* values refer to the comparisons among the three groups. Different superscript letters indicate significant difference between groups. Values of *p* < 0.05 were considered significant (italics)

### Pain and sedation assessments

Sedation scores were higher at 0.5 h [4 (2–5); *p* < 0.0001] in IV group, at 0.5 h [4 (2–10); *p* < 0.0001] and 1 h [2 (0–4); *p* < 0.0001] in IM group, and at 0.5 h [2 (0–6); *p* < 0.0001] and 1 h [3 (2–4); *p* = 0.0004] in SC group when compared with baseline values [0 (0–0) for all groups]. There were no differences in DIVAS scores between groups except at the time-point 0.5 h when scores were higher in IM [4 (2–10)] when compared with SC [2 (0–6)] group (*p* = 0.0003).

**Fig. 2:**
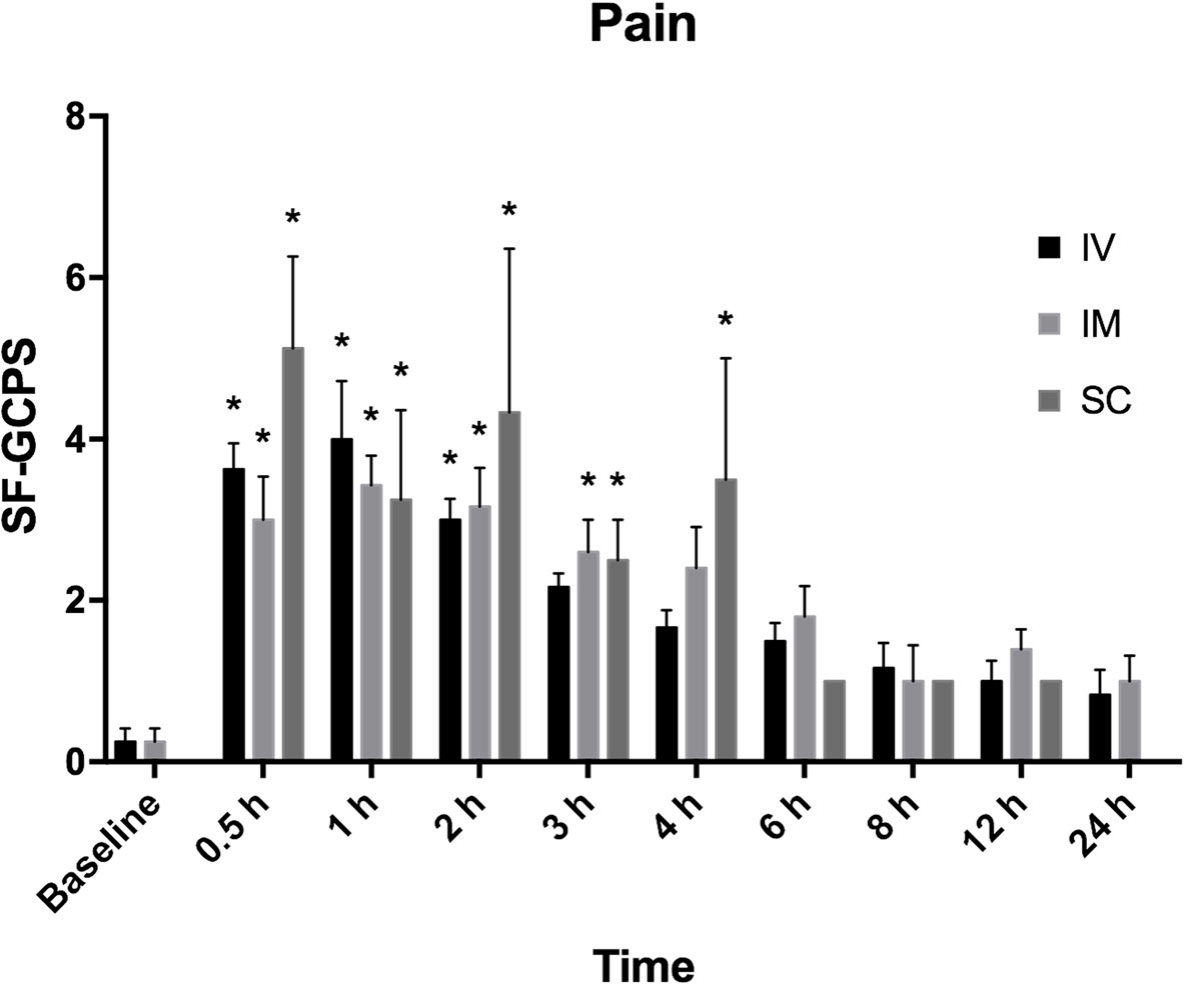
Mean and SD pain scores using the short-form Glasgow composite pain scale for dogs (SF-GCPS). The * represents significant differences when compared with baseline. There is no bar for SC group at baseline and at 24 h because the mean value was equal to zero. There are no error bars for SC group at 6, 8 and 12 h because data were available for a single dog only (after exclusion of pain score data from other dogs due to administration of rescue analgesia). The number of dogs/group varies overtime since pain scores were excluded from analysis after the administration of rescue analgesia (Table 2)

Pain scores were higher at 0.5 h, 1 h and 2 h (*p* < 0.0001) in IV group, at 0.5 h, 1 h, 2 h (*p* < 0.0001) and 3 h (*p* = 0.0007) in IM group, and at 0.5 h, 1 h, 2 h (*p* < 0.0001), 3 h (*p* = 0.0005) and 4 h (*p* = 0.0002) in SC group when compared with baseline values (Fig. 2). There were no significant differences between groups at any time point (*p* > 0.002; not significant after adjustment).

Prevalence of rescue analgesia was significantly higher in SC (7/8 dogs) than IV (2/8) (*p* = 0.0406) but not different between IV and IM (3/8) (*p* = 1) or IM and SC (*p* = 0.1189) (Table 2). The frequency of rescue analgesia was not significantly different among groups (IV = 2, IM = 5 and SC = 9) (*p* = 0.07) (Table 3).

**Table 2:** Prevalence of rescue analgesia according to the route of administration and timepoints in dogs undergoing ovariohysterectomy and administered buprenorphine (Simbadol; 0.02 mg/kg) by the intravenous (IV), intramuscular (IM) or subcutaneous (SC) routes of administration

Route of administration	Baseline (hours)	Postoperative time points after extubation (hours)									Total
	0	0.5	1	2	3	4	6	8	12	24	
IV	8	1	1	0	0	0	0	0	0	0	2
IM	8	1	1	1	0	0	0	0	0	0	3
SC	8	4	1	1	0	1	0	0	0	0	7

**Table 3:** Number (frequency) of administrations of rescue analgesia in dogs undergoing ovariohysterectomy and administered buprenorphine (Simbadol; 0.02 mg/kg) by the intravenous (IV), intramuscular (IM) or subcutaneous (SC) routes of administration

Route of administration (*n* = 8/group)	Postoperative time points after extubation (hours)									Total
	0.5	1	2	3	4	6	8	12	24	
IV	1	1	0	0	0	0	0	0	0	2
IM	1	1	1	1	0	1	0	0	0	5
SC	4	1	1	2	1	0	0	0	0	9

### Disposition of buprenorphine and norbuprenorphine and pharmacokinetics

**Fig. 3:**
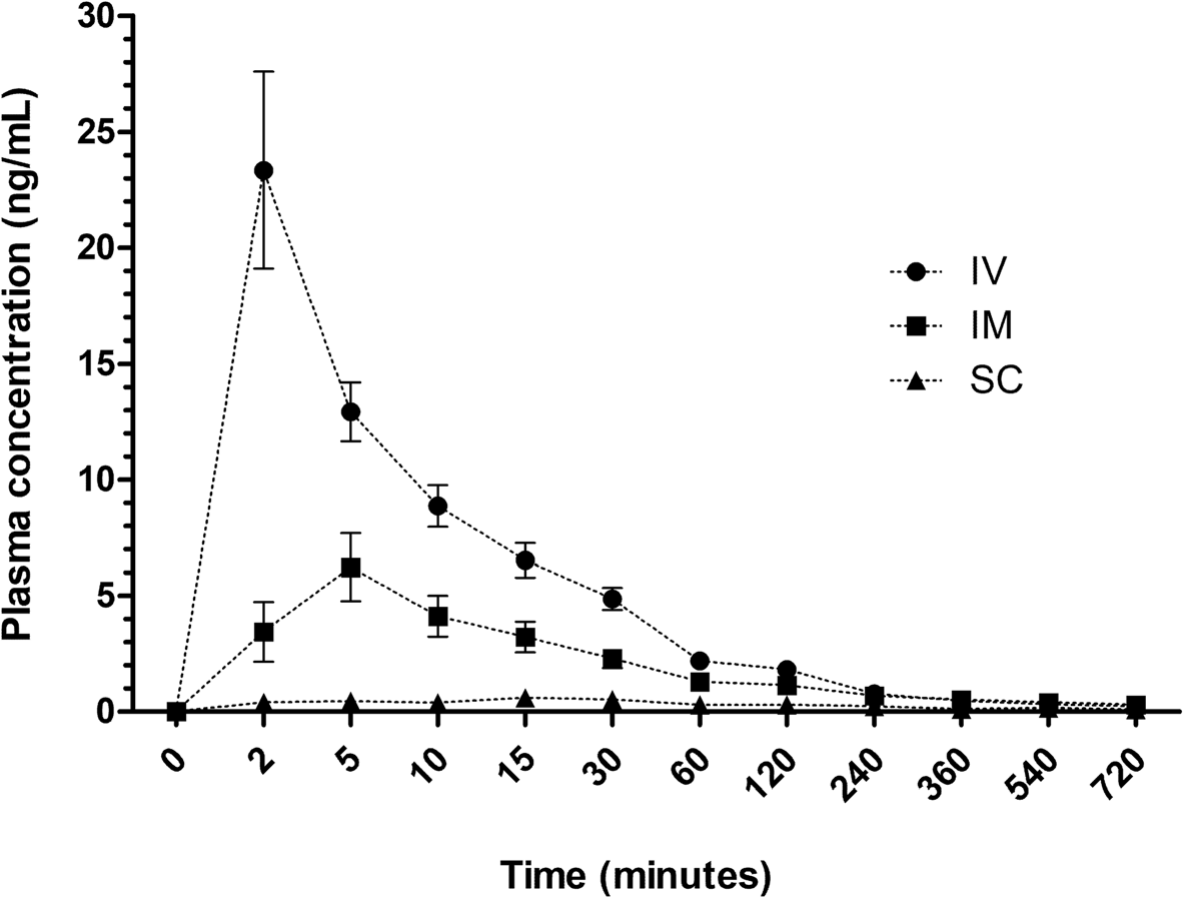
Mean ± SEM plasma concentrations (ng/mL) of buprenorphine (Simbadol) versus time (minutes) after intravenous (IV), intramuscular (IM) and subcutaneous (SC) administration (0.02 mg/kg) in dogs undergoing ovariohysterectomy (*n* = 8/group)

Plasma concentrations of buprenorphine versus time curves after IV, IM and SC administration are reported in Fig. 3. Concentrations of buprenorphine were quantifiable up to 720 min for all routes and dogs. There was large inter-subject variability in pharmacokinetic parameters after IV, IM and SC administration (Table 4). For the SC group, there was uncertainty about the slope of the elimination phase. The pharmacokinetic variables are reported in Table 4. Except for two dogs in the SC group, data were suitable for pharmacokinetic modelling for all other individuals. Norbuprenorphine was not measurable in any dog.

**Table 4:** Pharmacokinetic (PK) variables following intravenous (IV), intramuscular (IM) or subcutaneous (SC) administration of buprenorphine (Simbadol; 0.02 mg/kg) in dogs undergoing ovariohysterectomy. Data are reported as mean (95% confidence interval)

Parameter	Unit	IV (*n* = 8)	IM (*n* = 8)	SC (*n* = 6)*
AUC _0-t_	ng/mL*h	14.49 (12.86–16.11)	9.32 (6.53–12.11)	5.79 (4.26–7.32)
AUC _0-∞_	ng/mL*h	15.77 (13.87–17.67)	12.41 (10.39–14.43)	16.45 (8.00–24.90)
C_0_ or Cmax	ng/mL	35.92 (14.42–57.43)	6.24 (2.78–9.70)	1.37 (0.89–1.85)
*F*	%	–	62.6	40
T*max*	h	–	0.14 (0.01–0.26)	0.33 (0.20–0.47)
λ z	1/h	0.19 (0.17–0.21)	0.14 (0.10–0.18)	0.04 (0.02–0.06)
t_1/2_	h	3.69 (3.25–4.14)	5.66 (2.82–8.50)	21.95 (12.03–31.87)
MRT_0-∞_	h	3.59 (3.06–4.12)	7.37 (3.00–11.73)	30.02 (15.77–44.27)
Cl or Cl/F	L/h/kg	1.29 (1.14–1.45)	1.65 (1.40–1.91)	1.40 (0.91–1.88)
V_z_ or V_z_/F	L/kg	6.82 (6.06–7.58)	14.16 (4.50–23.81)	40.13 (26.27–53.98)
V_ss_	L/kg	4.59 (3.98–5.19)	NC	NC

* The PK of buprenorphine could not be derived in two dogs after subcutaneous administration due to erratic drug absorption. AUC, area under the plasma concentration-time curve from zero to infinity (_**0-∞**_) or to the last measurable concentration (_**0-t**_); MRT, mean residence time; Cl or Cl/F, plasma clearance or apparent clearance; t_1/2_, elimination half-life; λ z, elimination rate constant; *F*, bioavailability; V_z_ or V_z_/F, apparent volume of distribution at pseudo equilibrium during the elimination phase; Tmax, time to maximal concentration; Cmax, maximal serum concentration; V_ss_, apparent volume of distribution at steady state; NC, non calculable

Approximate plasma concentrations of buprenorphine at the time of rescue analgesia were as follows (ng/mL; # patient number): 1.33 (# 1) and 1.87 (# 24) in the IV group; 0.18 (# 2), 0.42 and 0.40 (# 8) and 1.24 and 0.76 (# 9) in the IM group, and 0.20 (# 5), 0.38 (# 6), 0.24 and 0.35 (# 10), 0.72 and 0.41 (# 11), 0.54 (# 14), 0.46 (# 21), 0.25 (# 25) in the SC group. Mean ± SD approximate plasma concentrations of buprenorphine (ng/mL) at the time of rescue analgesia were 1.60 ± 0.38, 0.60 ± 0.41 and 0.39 ± 0.17 for the IV, IM and SC groups, respectively.

## Discussion

The route of administration influences the PK, postoperative pain scores and prevalence of rescue analgesia after buprenorphine (Simbadol) in dogs undergoing ovariohysterectomy. Pain scores returned to baseline values earlier in the IV group (3 h) followed by the IM (4 h) and the SC group (6 h), besides the lack of significance when scores were compared between groups. Additionally, the prevalence of rescue analgesia was significantly smaller in the IV when compared with the SC group, but not between the IV and IM nor the IM and SC groups. It may be hypothesized that this lack of difference between IM and SC groups could be related to a type II error due to the small sample size with a potential clinically relevant analgesic effect. Furthermore, pain scores were not significantly different between groups because scores were excluded from statistical analysis after rescue analgesia. This approach may overestimate the analgesic effects of a treatment since higher pain scores could have been possibly omitted from analysis introducing selection bias. However, this approach avoided analysis bias and should not be a problem when prevalence of rescue analgesia was used as an important study outcome. Overall, these findings could indicate a superior analgesic efficacy after IV than IM and SC buprenorphine in dogs. On the other hand, except for earlier return to baseline pain scores in the IV group, the results do not allow to make a clear distinction between the analgesic effects of IV and IM buprenorphine. Indeed, the administration of IM buprenorphine (Simbadol) with carprofen produced satisfactory analgesia in dogs undergoing ovariohysterectomy in a similar clinical setting [4].

A similar effect of route of administration was observed in cats. The IV or IM administration of standard concentrations of buprenorphine (0.3 mg/mL) provided greater antinociception or analgesia than the buccal and SC routes in both clinical and experimental settings [9, 10]. The current study used a different concentration of buprenorphine (1.8 mg/mL). However, this should not impair the rate of buprenorphine’s diffusion across membranes since the same active ingredient, dose and body location for injections were used. According to previous research in cats using different concentrations of buprenorphine but similar doses, maximum plasma concentrations could be influenced by drug concentration but not time to peak effect or analgesia [11]. The effect of route of administration on analgesia could be then explained by the resulting PK in this study. The IV and IM concentration-time profiles decreased curvilinearly after the administration of buprenorphine showing classical distribution and elimination. On the other hand, buprenorphine uptake and plasma concentrations were very low after SC administration with longer t_1/2_ and MRT, and smaller AUC in comparison with the IV and IM routes. The wide range of t_1/2_ and MRT values may reflect differences in the extent of drug redistribution and possible cytochrome P450 polymorphisms since Cl was similar among groups. It has been hypothesized that the resulting low concentration gradient after SC administration does not allow buprenorphine to reach effective concentrations at the opioid receptor level resulting in poor analgesic efficacy in cats [9] similarly to the findings in this study. In the IV and IM groups, plasma concentrations of buprenorphine were likely enough to allow the drug to transfer down its concentration-gradient leading to the occupation of opioid receptors and producing a better analgesic effect than the SC group. For example, mean C_max_ was only 1.37 ng/mL in the SC group whereas it reached 6.24 ng/mL in the IM group with a mean T_max_ of approximately 20 and 8 min, respectively.

There is usually evidence of hysteresis with a time-lag between the peak plasma concentrations and maximum antinociceptive or analgesic effects after the administration of buprenorphine in dogs and cats using different routes of administrations [3, 8, 9]. The plasma concentrations of buprenorphine correlated with clinical analgesia have not been clearly demonstrated. In one study, it was suggested that plasma concentrations of buprenorphine should be higher than 0.6 ng/mL for therapeutic efficacy following canine soft tissue surgery since many dogs required rescue analgesia at these concentrations [12]. However, another study found that dogs required rescue analgesia even with concentrations at 2 ng/mL [13]. In the present study, two dogs in the IV group and one dog in the IM group required rescue analgesia when concentrations were higher than 1 ng/mL. On the other hand, all dogs requiring analgesia in the SC group had concentrations lower than 1 ng/mL. Finally, some dogs had low pain scores when concentrations were lower than 1 ng/mL within the first 2 h postoperatively. This demonstrates the complex relationship between plasma concentrations and analgesic effects of buprenorphine where individual variability in PK and potentially pharmacodynamic effects may play a major role in the clinical setting.

The pharmacokinetic parameters estimated in the current study are somehow similar to those previously reported in the literature with IV buprenorphine in dogs. For example, the AUC_0-∞_, the λ z, Vss and MRT values for the IV group were similar to a recent publication [6] but differences exist to previously reported kinetics [7]. Nonetheless, comparisons related to the PK are difficult because of individual variability, study design (time points for blood collection, number of subjects, doses, route of administration, drug formulations), reporting units, analytical method, type of compartmental analysis and the effects of anesthetics and surgery. To the authors' knowledge, the PK of IM buprenorphine have not been reported making comparisons even more difficult. Therefore, PK parameters are better considered using different routes of administration in a comparative manner as presented herein.

Besides a classical disposition and elimination of buprenorphine, some dogs in the IV and IM groups still required rescue analgesia. It is not known if these animals had low nociceptive thresholds (more sensitive to the surgical trauma) and they would have required rescue analgesia regardless of the type of opioid used, or if indeed buprenorphine was not the best choice for these patients and a full agonist should have been used in place. For instance, methadone has been shown to provide superior analgesia to buprenorphine in dogs undergoing ovariohysterectomy [1]. In face of high pain scores required for rescue analgesia, some clinicians could opt to give a second dose of buprenorphine at a risk that the drug would not provide additional analgesia. In a study in dogs, a second dose of buprenorphine 4 or 6 h apart did not significantly reduce pain scores or nociceptive thresholds in dogs undergoing routine castration [14]. Increasing the doses of buprenorphine from 20 to 40 μg/kg did not provide additional analgesia in dogs undergoing ovariohysterectomy [15]. An alternative option for rescue analgesia would be to administer a full agonist of μ-opioid receptors (i.e. morphine) also at a risk that the negative interaction between morphine and buprenorphine at the opioid receptor level would reduce the overall analgesic effects as demonstrated with sufentanil and buprenorphine in dogs [16]. The authors opted for the second approach and to use morphine for rescue analgesia. Most importantly, pain assessment was continuously performed to ensure patient comfort and to confirm morphine had been effective. However, it is possible that some dogs required a second dose of morphine because the interaction between morphine and buprenorphine produced less than optimal analgesic effects.

Norbuprenorphine was not detected in the plasma of any dog similarly to the observations of previous studies [8, 17]. In cats, the kinetic of norbuprenorphine followed closely the kinetics of buprenorphine, however the contribution of norbuprenorphine to thermal antinociception could not be elucidated [3]. Norbuprenorphine is between 50 and 200-fold less potent than its parent compound in terms of respiratory and antinociceptive effects [18] and it is not known how it contributes to the overall clinical analgesic effects of buprenorphine in dogs. Dogs produce small concentrations of this metabolite which are usually below the limits of quantification using LC-MS/MS [17].

Dogs in the SC group were younger (1.7 ± 1.8 years) and received significant higher doses of propofol (5.3 ± 1.0 mg/kg) when compared with IV group (4.6 ± 2.5 years and 4.2 ± 0.8 mg/kg, respectively). It is difficult to predict the effects of these findings on the PK of buprenorphine, postoperative pain scores and prevalence of rescue analgesia. However, considering that doses of propofol were still within the range used in clinical practice in both groups, it is possible that IV buprenorphine produced better sedation and some propofol-sparing effect when compared with the SC group. Additionally, there were two 8-year old and two 7-month old dogs in the IV and SC groups, respectively, contributing to the age difference between groups. In these patients, it is possible that the V_z_ of propofol was different between groups and higher anesthetic requirements were needed in younger patients.

Sedation scores were significantly increased in the early postoperative period as residual anesthetic effects slowly subsided in all groups. It is not known exactly how sedation affects pain assessment; therefore, it is important to demonstrate that sedation did not bias pain scores and that DIVAS scores were similar among groups throughout the study. At 0.5 h, DIVAS scores were higher in the IM than in the SC group. These low levels of sedation in the SC group could demonstrate pain-induced restless and agitation since four dogs required rescue analgesia at 0.5 h in this group. Clinically, it shows that signs of pain can be observed as early as 0.5 h after extubation in dogs undergoing ovariohysterectomy and pain assessment should not be delayed due to the residual effects of anesthetics.

Some limitations have been already discussed. The study did not involve the administration of standard concentrations of buprenorphine (0.3 mg/mL) that could elucidate potential differences in kinetics when compared with Simbadol (1.8 mg/mL). The study design also involved only one dose of buprenorphine. It is not known if increasing doses of buprenorphine would provide better postoperative analgesia. In cats, an approximate 10-fold increase in dose (0.24 mg/kg) resulted in thermal antinociception for up to 30 h when SC buprenorphine was administered [3]. Finally, the study involved two classes of analgesics (a nonsteroidal anti-inflammatory drug and an opioid). Recently, the use of intraperitoneal analgesia has been recommended by the World Small Animal Veterinary Association Global Pain Council in a review [19]. This simple, cost-effective technique could be used concomitantly and potentially improve pain management as part of multimodal analgesia.

## Conclusion

The PK of IV and IM buprenorphine followed classical disposition and elimination in dogs. The route of administration influenced the analgesic efficacy of buprenorphine in dogs which could be explained by erratic drug absorption after SC administration. Pain scores returned earlier to baseline values in the IV than in the IM and SC routes of administration. The prevalence of rescue analgesia was significantly lower in the IV than in the SC group.

At the doses used and based on pain scores, prevalence of rescue analgesia and plasma concentrations, buprenorphine (Simbadol) should not be administered for postoperative analgesia by the SC route in dogs undergoing ovariohysterectomy even when administered in combination with carprofen; the IV and IM routes should be preferred.

## Methods

### Study design

The study protocol was approved by the *Comité d’éthique de l’utilisation des animaux* of the Faculty of Veterinary Medicine, Université de Montréal (18-Rech-1979). This study is reported according to the Consolidated Standards of Reporting Trials (CONSORT) guidelines for randomized clinical trials [20]. This was a prospective, randomized, masked clinical trial.

### Animals

Twenty-six female dogs from local animal shelters were admitted to the veterinary teaching hospital (*Centre Hospitalier Universitaire Vétérinaire*) of the Faculty of Veterinary Medicine, Université de Montréal for elective ovariohysterectomy between May and July 2019. Written consent for participation in the study was obtained for each patient. Dogs were included if they were considered healthy based on medical history, physical examination, hematology and biochemical panel. They were up to date on vaccination and parasite control. Dogs of any breed with age between 6 months and 8 years and weighting between 5 and 30 kg were included. Patients with aggressive or fearful behaviors, pregnancy or any sign of disease were excluded. Dogs were admitted the day before surgery and were housed individually in a dog ward. Each run was equipped with blankets, water and food bowls. Food but not water was withheld for 8–12 h. At the end of the study, dogs returned to their local shelters for adoption.

### Anesthesia, surgery and treatments

Randomization of treatments was performed by an individual not involved with pain assessment using a randomization plan generator (www.randomization.com). Upon arrival, each dog was sequentially assigned a number (1–24). According to this number, the patient was allocated to one of the three treatment groups (*n* = 8/group). If any dog from 1 to 24 was excluded, additional dogs were recruited and given the same treatment of the excluded dog on the order of arrival.

After physical examination and baseline pain assessment, an IV catheter was placed aseptically in each cephalic vein. Dogs were then randomly premedicated with 0.02 mg/kg of buprenorphine (Simbadol) via intravenous (group IV), intramuscular (group IM) or subcutaneous (group SC) route of administration. The IM administration was performed into the epaxial muscles. The SC administration was performed between the scapulae. The two catheters were color-coded: the ones wrapped in blue bandage were used for drug and fluid administration (including premedication in the IV group) and were removed 6 h after surgery. The catheters wrapped in red bandage were used exclusively for blood sampling and removed following collection of the last blood sample.

Thirty minutes after premedication with buprenorphine, anesthetic induction was accomplished with intravenous propofol (PropoFlo 28, Zoetis, Canada) until endotracheal intubation was possible using an appropriately sized cuffed endotracheal tube. General anesthesia was maintained with isoflurane (Isoflurane USP, Fresenius Kabi, Canada) in oxygen. Following induction of anesthesia, carprofen was administered (4.4 mg/kg SC; 50 mg/mL, Rimadyl, Zoetis, Kirkland, QC, Canada) and dogs were positioned in dorsal recumbency over a circulating warm-water blanket. A balanced crystalloid solution (Lactated Ringer’s solution USP, Baxter, Canada) was administered (10 mL/kg/h) throughout surgery. Anesthetic monitoring was performed using a multi-parametric monitor (Lifewindow 6000 V, Digicare Animal Health, USA) with electrocardiography, non-invasive blood pressure via the oscillometric method, capnography, inspired and expired concentrations of isoflurane, pulse oximetry and esophageal temperature. Ovariohysterectomy was performed by the same veterinarian using a midline approach and a two-clamp modified technique. The abdominal wall and subcutaneous tissues were closed with a simple continuous pattern of absorbable suture material whereas the skin was closed using an intradermal suture pattern. Surgery time (time elapsed from the first incision until placement of the last suture), anesthesia time (time elapsed from injection of propofol to turning off the vaporizer dial) and time to extubation (time elapsed from turning off the vaporizer dial until extubation) were recorded. The length of incision was recorded after skin closure. A 2-cm green line tattoo was applied laterally to the ventral midline incision for visual identification of a neutered dog. Dogs recovered from anesthesia in a warm and quiet environment. An additional dose of carprofen (4.4 mg/kg orally) was administered 24 h after the first dose.

### Pain and sedation assessments

Assessments were performed by an observer with previous experience in pain assessment who was blinded to the routes of administration. Pain was evaluated using the short-form Glasgow composite pain scale for dogs (SF-GCPS) [21]. This scale includes 30 descriptor options within six behavioral categories. Within each category, the descriptors are ranked numerically according to their associated pain. In this study, section “B” of the SF-GCPS (“put lead on dog and lead out of kennel”) was not performed since dogs were unlikely to stand up and walk shortly after anesthesia. Thus, the maximum total score of the SF-GCPS was 20 [21]. The SF-GCPS evaluations were performed 1 h before anesthetic induction (time 0 h, baseline) and at 0.5, 1, 2, 3, 4, 6, 8, 12 and 24 h after extubation. Sedation was evaluated using a dynamic and interactive visual analog scale (DIVAS) where 0 was considered as no sedation and 100 mm as the maximum sedation where dogs would be in lateral recumbency and not respond to stimulation [22]. Rescue analgesia was performed if SF-GCPS scores were ≥ 5/20 using morphine which was administered over 1 min (0.25 mg/kg IV 10 mg/mL, Morphine Sulfate Injection, Sandoz Canada Inc., Boucherville, QC, Canada). Data collected after the administration of rescue analgesia were not included in the statistical analysis; however, dogs were continuously monitored for additional need of analgesics. The number of dogs, timing of rescue administration and number (frequency) of rescue treatments were recorded.

### Blood collection

This was performed just before and at 2, 5, 10, 15, 30, 60, 120, 240, 360, 540 and 720 min after the administration of buprenorphine [5, 7]. One individual was responsible for blood collection while another observer was responsible for pain assessment. The volume of blood collected was adjusted for each individual so that less than 10% of the dog’s total blood volume was removed over the study period (2 mL maximum per sample). For each sample, a total of 0.3–0.5 mL of blood was first collected and discarded to avoid contamination from a previous time point. A second syringe was then used to collect the blood sample for analysis, which was immediately transferred to an EDTA K3 tube and stored in ice. The catheter was then flushed with 1 mL of heparinized saline solution. The blood sample was then centrifuged at 2000 *g* for 10 min. Plasma was separated and stored at − 80 °C until buprenorphine assay was performed.

### Buprenorphine assay

The concentrations of buprenorphine and norbuprenorphine were determined in plasma using a validated liquid chromatography with tandem mass spectrometry method (HPLC-MS/MS). Detailed methodology, and test precision and accuracy are described in Additional file 1.

### Pharmacokinetic analysis

Buprenorphine plasma concentrations versus time data for each patient were plotted and visually inspected. The pharmacokinetic analysis of buprenorphine was performed using a standard software (PK Solver 2.0). Non-compartmental analysis was performed for the three routes of administration. The area under the time-concentration curve from time zero to infinity (AUC _0-∞_) and area under the curve to the last measurable concentration (AUC _0-t_) were calculated by use of a log-linear trapezoidal model. Standard equations were used to calculate other estimates including elimination half-life [t (1/2)], plasma clearance (Cl), apparent volume of distribution at steady state [V_z_ (ss)], and mean residence time (MRT). Time to maximal concentration [T (max)] and maximal serum concentration [C (max)] were taken directly from the data [5, 7].

### Statistical analysis

The study would require eight dogs per group to detect a difference of 3 points in pain scores between two means (IV and IM) using the SF-GCPS and considering an alpha value of 5%, a power of 80% and a standard deviation within group of 2 points. These values were based on clinical experience where changes in 3 points in SF-GCPS would likely reflect clinically relevant changes in patient comfort. Statistical analyses were performed with SAS (version 9.3; SAS Institute, USA) and figures were plotted with GraphPad Prism 8 (version 8.0.2; GraphPad Software Inc., USA). Demographic data between groups were analyzed using linear models with group as fixed factor. The prevalence of rescue analgesia was analysed using Fisher’s exact test for each comparison between groups. The number (frequency) of rescue analgesia was analysed using the Mantel-Haenszel chi-square test for ordinal variables. Pain and sedation scores were analysed using a linear mixed model with group as between-subject fixed factor, time as a within-subject fixed factor and dog as the random effect. A series of a priori contrasts were performed to compare scores within- and between-groups. The Benjamini-Hochberg sequential procedure was used for adjustment after multiple comparisons. Significance was set at α < 0.05.

## Supplementary information

**Additional file 1.** : Analytical method (HPLC-MS/MS) for buprenorphine and norbuprenorphine after intravenous, intramuscular and subcutaneous administration in dogs undergoing ovariohysterectomy

## Data Availability

The datasets used and/or analyzed during the current study are available from the corresponding author on reasonable request.

## Abbreviations

PK: Pharmacokinetics
SC: Subcutaneous
IM: Intramuscular
IV: Intravenous
SF-GCPS: Short-form Glasgow composite measure pain scale
DIVAS: Dynamic and interactive visual analog scale
AUC: Area under the plasma concentration-time curve from zero to infinity (_**0-∞**_) or to the last measurable concentration (_**0-t**_)
*F*: Bioavailability
MRT: Mean residence time
Cl or Cl/F: Plasma clearance or apparent clearance
t_1/2_: Elimination half-life
V*z*: Apparent volume of distribution at pseudo equilibrium during the elimination phase
V_ss_: Apparent volume of distribution at steady state
NC: Non calculable

## Footnotes

1 https://atwork.avma.org/2018/02/16/avma-working-group-helps-navigate-opioid-abuse-epidemic/
