# Supplementary material for: Pharmacokinetics and analgesic effects of intravenous, intramuscular or subcutaneous buprenorphine in dogs undergoing ovariohysterectomy: a randomized, prospective, masked, clinical trial
Source: BMC Vet Res. 2020 May 24;16:154. doi: 10.1186/s12917-020-02364-w (PMC7245774; doi:10.1186/s12917-020-02364-w)
Supplement: Supplementary file 1 — Additional file 1. : Analytical method (HPLC-MS/MS) for buprenorphine and norbuprenorphine after intravenous, intramuscular and subcutaneous administration in dogs undergoing ovariohysterectomy [file 12917_2020_2364_MOESM1_ESM.docx]

**Analytical method (LC-MS/MS) for buprenorphine and norbuprenorphine after intravenous, intramuscular and subcutaneous administration in dogs undergoing ovariohysterectomy**

# Analytical Procedure

### Reagents

Buprenorphine, norbupreno rphine, ^2^H4-buprenorphine, ^2^H3- norbupreno rphine were purchased Cerilliant (Round Rock, TX, USA). Drug free <log plasma was supplied by the laboratory of Dr. Steagall. Formic acid was purchased from Sigma Aldrich (St-Louis, MO, USA). Other chemicals, including, methanol, acetonitrile, ammonium hydroxide, ethyl acetate and water were purchased from Fisher Scientific (Fair Lawn, NJ, USA).

### Sample preparation

Dog plasma samples from Dr. Paulo Steagall study were analyzed for buprenorphine and norbuprenorphine by a liquid chromatography-mass spectrometry (LC-MS/MS) method. Using a liquid-liquid extraction as sample preparation technique, buprenorphine and norbuprenorphine were extracted from dog plasma. Two hundred and fifty microliters of interna! standard solution (10.0 ng/mL of ^2^H4- buprenorphine and ^2^H3- norbupreno rphine in water) was added to an aliquot of one hundred and fifty microliters of sample and subsequently alkalinized using 50 µL 5 M ammonium hydroxide solution in a 16 x 100 mm borosilicate screw cap tube. The sample was vortexed for approximately 5 seconds. Four mL of ethyl acetate was added to the sample and gently mixed by rotation for 20 minutes. The sample was then centrifuged at approximately 3500 *g* for 10 min and the organic layer was transferred into a clean 13 x 100 mm borosilicate tube and evaporated to dryness at 50°C under a gentle stream of nitrogen. The dried extract was resuspended with 75 µL of 20:80 (v/v) methanol:water solution and transferred to an injection vial for analysis.

### Chromatographie conditions

An isocratic mobile phase was used with a Phenomenex PFP(2) (150 x 3 mm I.D., 3 µm) and PFP (4 x 2.0 mm) security guard cartridge operating at 50°C. The mobile phase conditions consisted of acetonitrile and 1.0% (v/v) formic acid in water at a ratio of 50:50, respectively. The flow rate was fixed at 0.30 mL/min and norbuprenorphine, buprenorphine and their respective interna! standards (2H3-norbuprenorphine and ^2^H4- buprenorphine) eluted at 3.5 and 4.9 minutes, respectively. Ten microliters of the extracted sample was injected and the total run time was set at

8.0 minutes.

### 1.4 Mass spectrometric conditions

The mass spectrometer was interfaced with the UPLC system using a pneumatic assisted heated electrospray ion source. MS detection was performed in positive ion mode, using selected reaction monitoring (SRM). In order to optimize the MS/MS parameters, standard solutions of buprenorphine and norbuprenorphine were infused into the mass spectrometer. The following parameters were obtained. Nitrogen was used for the sheath and auxiliary gases and was set at 50 and 15 arbitrary units. The HESI electrode was set to 3500 V. The capillary temperature was set at 350°C. Argon was used as collision gas at a pressure of 2.5 mTorr. The precursor-ion reactions and the collision energy for buprenorphine, norbuprenorphine and their respective interna! standards are in table 1. Total cycle time was set at 0.25 seconds. Peak width of Ql and Q3 were both set at 0.7 FWHM.

#### Table 1: Precursor ion reactions and Collision energies for buprenorphine, norbuprenorphine 2H4-buprenorphine and 2HJ-norbuprenorphine

| Compound | Precursor (m/z) | Product (m/z) | Collision Energy (V) | RF Lens (V) |
| --- | --- | --- | --- | --- |
| Norbuprenorphine | 414.4 | 187.1 | 38 | 85 |
| Norbuprenorphine | 414.4 | 340.3 | 31 | 85 |
| 2H3- norbuprenorphine | 417.4 | 343.3 | 38 | 85 |
| Buprenorphine | 468.4 | 396.3 | 40 | 85 |
| Buprenorphine | 468.4 | 414.3 | 35 | 85 |
| 2H4- buprenorphine | 472.4 | 400.3 | 40 | 85 |

# Analytical Qualification

Stock solutions were received in ampoules as certified solutions containing 1.0 mg/mL in methanol of buprenorphine and norbuprenorphine and 0.1 mg/mL in methanol for ^2^H4- buprenorphine and ^2^H3-norbuprenorphine. A series of standard working solutions of buprenorphine and norbuprenorphine were obtained by mixing the standard stock solution and further diluting with methanol. Calibration standards were prepared by fortifying the dog plasma with the standard working solutions at 2% (v/v) to enable concentrations spanning the following analytical range 0.10 to 100 ng/mL for buprenorphine and norbuprenorphine. The method is linear using a linear regression weighted 1/x analysis. R^2^ 0.9983 and 0.9965 for buprenorphine and norbuprenorphine (figures 2 and 3).

#### Figure 2: Buprenorphine calibration curve in dog plasma


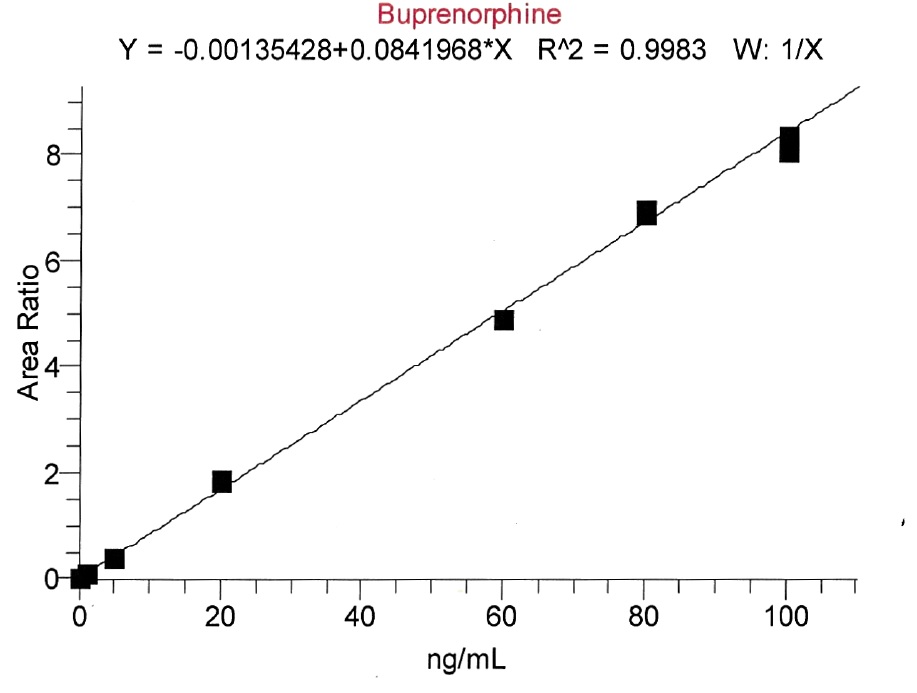


#### Figure 3: Norbuprenorphine calibration curve in dog plasma


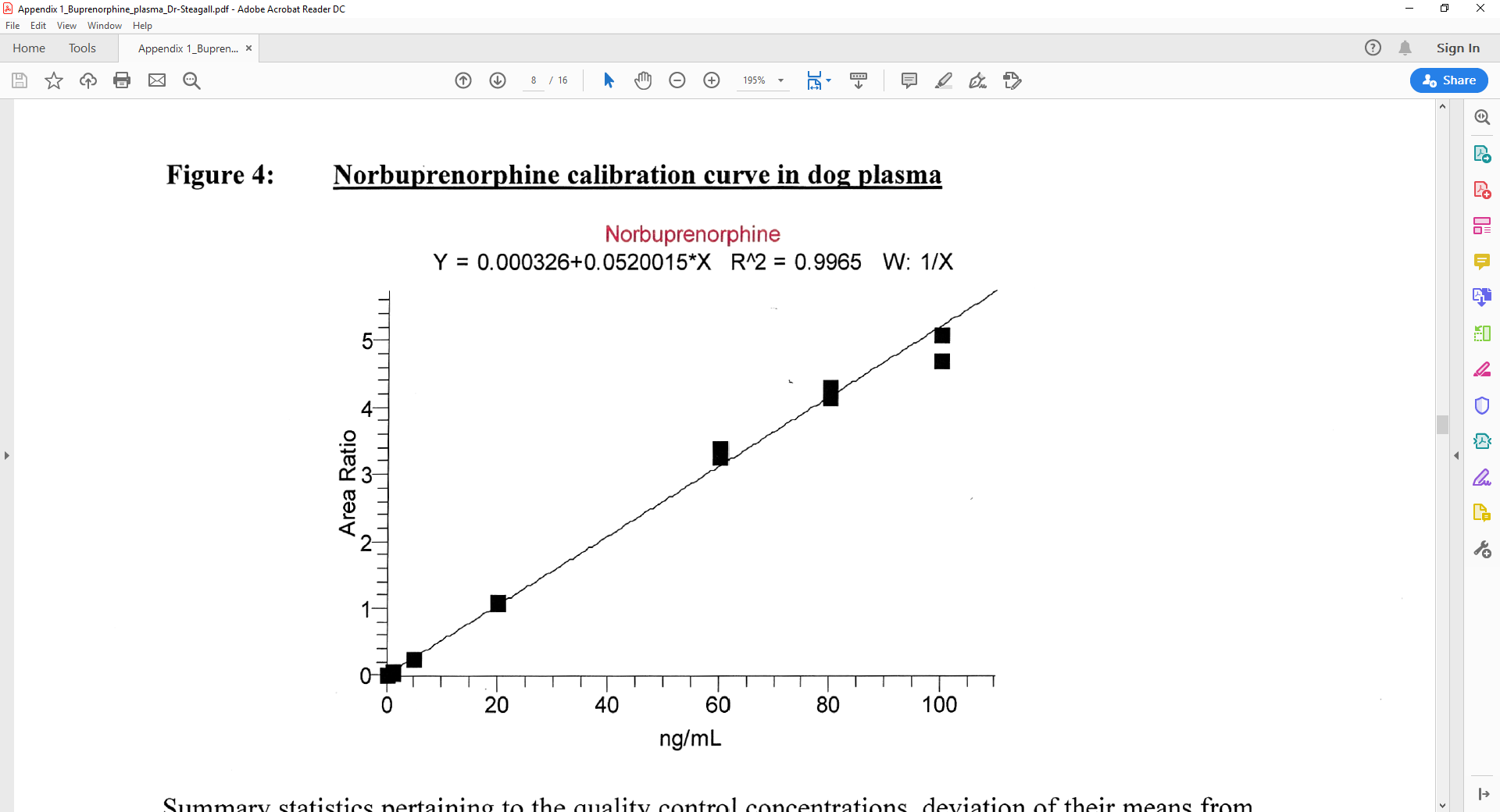


Summary statistics pertaining to the quality control concentrations, deviation of their means from the nominal concentrations, and composite results are shown in table 2.

#### Table 2: Summary of quality control concentrations of buprenorphine and norbuprenorphine in dog plasma during qualification

| Compound | Concentration | | Precision and Accuracy (n= 5) | | | |  |
| --- | --- | --- | --- | --- | --- | --- | --- |
|  | (ng/mL) | | mean ± SD (ng/mL) RSD (%) | | | | RE(%) |
|  | | 0.30 | 0.29 | ± | 0.012 | 4.0 | -2.3 |
| Buprenorphine | | 5.00 | 4.92 | ± | 0.102 | 2.1 | -1.7 |
|  | | 60.0 | 59.90 | ± | 1.4 | 2.3 | -0.2 |
|  | | 0.30 | 0.30 | ± | 0.0 | 4.7 | -0.8 |
| Norbuprenorphine | | 5.00 | 4.76 | ± | 0.1 | 2.0 | -4.8 |
|  | | 60.0 | 65.06 | ± | 0.6 | 1.0 | 8.4 |

RSD = Relative Standard Deviation, RE = Relative Error

# Sample Analysis

## During qualification, the method met all requirements of sensitivity, linearity, precision and accuracy within a batch. This assay is suitable for the analysis of buprenorphine and norbuprenorphine in dog plasma. The correlation coefficient for buprenorphine and norbuprenorphine during all sample analysis batches was greater than R2 2: 0.9945 and 0.9940 for buprenorphine and norbuprenorphine, respectively.
